# The Impact of Parental Involvement on the Educational Development of Students with Autism Spectrum Disorder

**DOI:** 10.3390/children11091062

**Published:** 2024-08-30

**Authors:** José Fernández Cerero, Marta Montenegro Rueda, Eloy López Meneses

**Affiliations:** 1 Department of Teaching and Educational Organization of Sevilla, 41013 Sevilla, Spain; jfcerero@us.es (J.F.C.); mmontenegro1@us.es (M.M.R.); 2 Department of Educational and Social Psychology, Pablo de Olavide University, 41013 Sevilla, Spain

**Keywords:** family, students, ASD, education, motivation

## Abstract

Background/Objectives: Parental involvement is vital for the academic success and overall development of students, particularly those with Autism Spectrum Disorder (ASD), who face unique educational challenges. This study investigates the influence of parental involvement on the academic performance and school adjustment of students with ASD, focusing on the interplay of cultural identity and first language in their educational and social growth. The research also seeks to identify the challenges parents face and the strategies they use to support their children. Method: Employing a qualitative, interpretive approach, this study involved 42 parents of secondary school students with ASD in Seville, Spain. Data collection included in-depth interviews and focus group discussions, allowing for a comprehensive understanding of parental experiences. Results: The study found that active parental involvement greatly enhances students’ academic performance and emotional well-being. Key strategies include creating structured home environments, utilizing assistive technologies, and fostering open communication with school staff. However, parents reported significant challenges, including limited school resources, communication barriers with educators, and a lack of ASD-specific training for teachers and parents. These challenges often hinder their ability to fully support their children’s education. Conclusions: Active parental involvement is crucial for the educational success of students with ASD. The findings highlight the need for improved communication between schools and families, increased resources, and targeted ASD training for both teachers and parents. Additionally, the study advocates for greater investment in assistive technologies to better support students with ASD. Addressing these needs could significantly improve the educational experiences and outcomes for these students.

## 1. Introduction

Inclusion and attention to diversity have become priority issues on educational agendas worldwide. Over the years, the approach has evolved significantly: initially focusing almost exclusively on students with disabilities, it has moved towards a more holistic approach that encompasses students with a wide range of needs [1,2]. Following this line, it is essential to take into account students with Autism Spectrum Disorder (ASD), as they are particularly vulnerable in educational settings that are not adequately adapted to their specific needs. These students can benefit significantly from personalised educational strategies, such as structured education, the use of assistive technologies, and specific training for educators in communication techniques and adaptive behaviour. 

ASD is a developmental disability that affects the social interaction, communication, and behaviour of individuals, influencing a large sector of the population. Epidemiological studies in Europe show that the prevalence of ASD is approximately 1 per 100 births [3]. We are faced with a student population that experiences educational disadvantage and is often on the verge of marginalisation and stigmatisation. This situation generates high levels of stress and anxiety in these students [4], which can profoundly affect their daily functioning and lead to problematic behaviours.

In this sense, parental involvement in their children’s education is a key factor in the academic success and all-round development of students, as there are mechanisms through which this involvement influences educational outcomes and factors that affect the level of parental involvement in the educational process. Such involvement, as explained by Belaić [5], falls into two broad categories: activities carried out at home and activities carried out at school. Involvement at home includes actions such as creating an appropriate study environment and participation in non-formal educational activities, while involvement at school refers to attendance at school meetings and events. In turn, proper parental involvement in education can significantly influence academic performance by helping to develop specific skills and enhance the understanding of knowledge [6].

Along these lines, parents of children with ASD face significant challenges related to managing behaviours that may be particularly challenging and communicating with their children. These difficulties include managing behaviours that may be disruptive and establishing effective communication [7]. Furthermore, Campilla et al. [8] point out that these behaviours can include hyperactivity, aggression, and repetitive behaviours, which further complicate daily interactions and family coexistence. These situations call for specific and often personalised strategies for each child that can address both the needs of the child and the family environment, promoting an atmosphere of mutual understanding and support. Research indicates that mothers, in particular, often face high levels of stress, anxiety and fatigue. This state of sustained stress and anxiety can significantly reduce parents’ quality of life, limiting their ability to function effectively both personally and professionally [9]. However, stigma and social misunderstanding of ASD can lead to loneliness and isolation for parents. Many parents experience judgement and lack support from family, friends, and the community. Furthermore, this insufficient support extends to the workplace, where they may face discrimination and a lack of flexibility [10,11]. Classroom diversity is a topic of growing interest in the field of education, reflecting the importance of creating inclusive and equitable learning environments that respond to the needs of an increasingly heterogeneous student population. Beyond understanding how this diversity is managed, it is crucial to explore the intersection of cultural identity and first language in the educational and social development of students, particularly those with Autism Spectrum Disorder (ASD). Linguistic and cultural identity, often grounded in one’s first language, plays a fundamental role in how these students interact and adapt to educational environments. Therefore, integrating this dimension into educational strategies is not only a response to an emerging research trend but also a practical necessity to foster more holistic development.

This expanded focus aligns with current research trends that emphasise the importance of considering socio-cultural and linguistic aspects in the education of students with ASD. Through an extended literature review and analysis of recent studies, this article aims to highlight how the conscious integration of cultural and linguistic identity can significantly improve inclusion and educational success for these students. Thus, we propose to offer a more comprehensive and detailed outlook that responds to current critiques and enriches our understanding of the impact of parental involvement in the educational development of students with ASD.

Parental involvement in the education of children with ASD manifests itself in a variety of ways, including attendance at school meetings, collaboration with teachers, and homework support at home. Several studies have highlighted that this involvement is correlated with better academic and social outcomes for children. In this regard, several studies have pointed out that parents of children with ASD tend to be more involved in their children’s education compared to parents of children who do not have any educational needs. These parents more frequently attend parent–teacher conferences and meet with school counsellors. However, they also express a higher degree of dissatisfaction with the communication and support provided by schools [12].

On the other hand, in terms of the relationships between parents and educational institutions, it should be noted that certain factors can significantly influence the level of parental involvement. Some of these factors such as family stress and parental well-being affect these interactions and, consequently, the effectiveness of parental involvement in the education of their children with ASD [13].

Following this line, the aim of the present study is to learn more about the impact of family involvement in the educational process of their children with ASD. To this end, the following research questions were explored:

RQ1: How does parental involvement and support influence the academic achievement of students with ASD?

RQ2: What family strategies and practises contribute most to the school motivation of students with ASD?

RQ3: What challenges do families of students with ASD face in their involvement and participation in the educational process?

RQ4: How do parents perceive communication and collaboration with the school in relation to the education of their children with ASD?

## 2. Methodology

### 2.1. Design

In order to achieve the established objective, a qualitative methodology based on an interpretative paradigm was adopted. This approach is ideal for social research, as it seeks to understand the specific context and meaning of the phenomena studied, as argued by Álvarez-Gayou [14]. In this sense, qualitative research allows us to learn more about whether parental involvement in the educational process of students with ASD has a positive or negative impact.

### 2.2. Sample

The sample of the present research is composed of 42 parents of secondary school students with ASD from various educational institutions in the province of Seville (Spain). The selection of the participants was established through a series of criteria: they must be parents or legal guardians of children who have been diagnosed with ASD; they must be attending secondary education in a public school in Spain; and they must be between 12 and 16 years old. The interviews were conducted between April and June 2024. A total of 64.2% of the students with ASD attending these studies were male and 35.7% were female. In addition, the students were aged between 12 and 16 years old. For the selection of the sample, convenience sampling was carried out, specifically by snowball sampling. As the vast majority of those interviewed had children in secondary education, it was decided to carry out the study focusing on the involvement of parents who have children in this educational stage. This method facilitated the identification of possible subjects within the target population. Considering the distribution of the sample according to gender, it can be observed that there is a very balanced participation, where 47.6% of the sample were men and 52.3% of the remaining sample were women, most of them with a university education. Table 1 shows the distribution of the sample according to the gender of the participants and the ownership of the school where their children are enrolled.

Families available to participate in the study were contacted by telephone between the months that the interviews were conducted. During these calls, informed consent was obtained from participants to include them in the research. In our study, the consideration of cultural factors was a priority to ensure the validity and relevance of our findings. First, we ensured that the study design and materials were culturally sensitive. This included reviewing and adapting the data collection tools by cultural experts so that they were understandable and respectful to participants from diverse cultural backgrounds. In addition, our research team was trained in cultural competency, focusing on understanding and respecting cultural differences that might influence participants’ responses.

### 2.3. Data Collection Instrument

In accordance with the instrument used in the present research, the semi-structured interview was applied as an instrument for collecting information. In addition to preliminary questions about the socio-demographic data of the participants, the interview script included ten questions focused on assessing and perceiving the impact of parents on the educational process of students with ASD. The interview script can be viewed in Appendix A.

To ensure the validity of the interview script, the Delphi method was implemented, selecting as a strategy the consultation of experts. This procedure was carried out by means of a document attached to the interview that included several open questions, with experts who have experience and in-depth knowledge in the integration of students with special needs. 

The Delphi method is a structured communication technique originally developed as a systematic, interactive forecasting method which relies on a panel of experts. In the context of this study, it was used to validate the interview script and ensure the relevance and comprehensiveness of the questions.

The Delphi method involves multiple rounds of questioning, with the following key steps:

- Selection of experts:

Experts were chosen according to two main criteria. Initially, specialists were identified who met two or more of the following requirements:

- Having published scientific articles on the inclusion of students with educational needs in the education system.
- Being trained in the educational needs of students.
- Having actively participated in research projects related to attention to diversity.
- Having taught in subjects related to attention to diversity.

- Initial questionnaire:

A comprehensive initial questionnaire was developed, including open-ended questions that covered all aspects of the interview script. Experts were asked to provide their feedback on the relevance, clarity, and comprehensiveness of the questions.

- First round of feedback:

The experts’ responses were collected and analysed. Common themes and suggestions for improvement were identified and used to refine the interview script.

- Subsequent rounds:

The refined questionnaire was then sent back to the experts for further review. This iterative process continued until a consensus was reached among the experts. This typically involved three to four rounds of feedback.

- Final consensus:

The final version of the interview script was agreed upon by all participating experts, ensuring that it was both comprehensive and relevant to the study’s objectives.

On the other hand, an unconventional method in educational research known as the “Expert Competence Coefficient” or “K-Coefficient” was used. It is a methodology used to select participants in educational research, especially when expert opinion is required. It is calculated using the formula: K = 1/2(Kc + Ka). Here, Kc represents the “Experience Coefficient”, which reflects the expert’s knowledge of the topic, and Ka is the “Argumentation Coefficient”, which evaluates the soundness of their reasoning [15]. The value of K ranges from 0 to 1, where a higher value indicates a higher competence of the expert. By using this method, researchers can ensure that they are selecting participants who not only have extensive knowledge of the topic, but are also able to provide well-supported arguments, which is crucial for the validity and reliability of the study. In our research, the K coefficient exceeded 0.8 for 11 of the 12 selected experts, indicating a highly satisfactory level of competence [16]. The evaluations by the experts were carried out in several anonymous rounds, following the Delphi method, to foster consensus while maintaining the independence of the participants.

### 2.4. Data Collection Procedure

The interviews were conducted remotely through telephone calls to ensure privacy and confidentiality, maintaining the anonymity of the participants throughout the process. Interviewers were given the opportunity to conduct the interviews via video call but the vast majority preferred to conduct the interviews by phone only. The adoption of this approach also made it possible to overcome geographical limitations, enabling the participation of parents of students from various educational institutions in the province of Seville (Spain). Each interview lasted approximately thirty minutes. All participants gave informed consent prior to their inclusion in the study.

Furthermore, the study was conducted in accordance with the Declaration of Helsinki [17], and the fundamental principles of research integrity were respected, in accordance with the Research Ethics Committee of the University of Seville.

### 2.5. Data Analysis Process

All telephone interviews were recorded with the prior consent of the participants. Subsequently, the recordings were manually transcribed and coded. The responses to the questions were organised into categories according to common criteria. The analysis of the information was carried out jointly by all the authors of the study. The analysis of the information followed several stages: recording of the interviews, transcription of the recordings, initial review of the transcripts, coding of categories and deductive subcategories (construction of the categorical system), and the creation of a semantic network for the macrocategory. For the content analysis, the software Atlas. Ti (2022) version 22.2.4 software was used. Table 2 shows the main categories and subcategories of the interview.

## 3. Results

**Figure 1:**
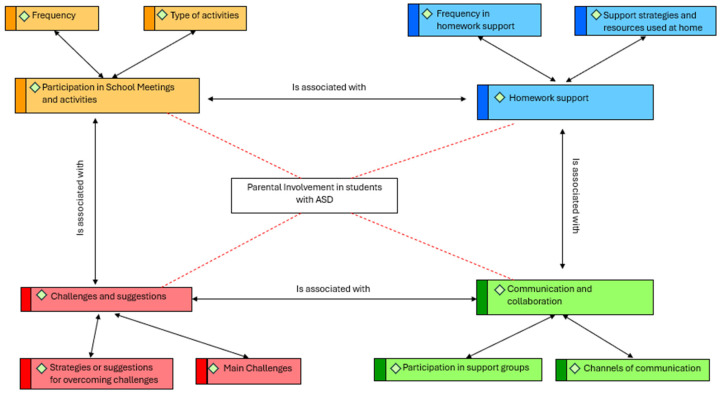
Graphical representation of macrocategories and subcategories in a semantic network.

Once the participants’ responses to the interviews had been analysed, their answers were categorised in relation to the objectives of the study and the categorisation previously carried out. It should be added that there were no significant differences in the results obtained according to the gender of the participants. Figure 1 shows the four categories and subcategories of the study that will be analysed below.

### 3.1. Participation in School Meetings and Activities

In order to gain a complete picture of family participation and involvement, it is crucial to analyse the interview responses for patterns that illustrate both the frequency of parental participation and the types of activities they attend. This analysis will help to better understand how and to what extent parents are involved in their children’s school life, identifying both strengths and areas that could be improved to encourage greater participation.

A total of 71.43% of the participants stated that they usually attend the activities and meetings that the educational institution organises, thus enabling them to have a better family–school relationship and to discuss academic and developmental issues of their children. That said, 28.57% stated that they do not attend the activities and meetings that the educational institution organises. The non-attendance or non-participation of these participants in these arranged meetings may be due to the lack of time or lack of interest in academic development, stating that this is the task of the school’s own teachers; however, they have also indicated that they are usually informed through e-mails provided to the school. Table 3 shows participation according to parents’ educational level.

“Personally, I often attend meetings convened by the school at least once every two weeks, as I am interested in knowing about updates and new proposals suggested both by other parents and by the school’s own teachers”.

(INTERVIEW 04)

“Sometimes, it is not possible for me to attend the meetings that the school organises to discuss problems that arise or simply to find out about our child’s academic performance, mainly because my work prevents me from doing so. My work schedule changes a lot, and I try to organise it with my husband, but sometimes it is not possible”.

(INTERVIEW 11)

As these results are strikingly similar to those of people with and without tertiary education, an analysis was carried out to see whether the breakdown of participation in school activities is associated with educational level, which may in turn influence flexibility in working hours, etc.

The data suggest a significant difference in participation according to parents’ level of education. Parents with higher education tend to participate more (attend) than those without higher education. To confirm whether these differences are statistically significant, the chi-square test was performed. The test score is 7.82 (sig. 0.0052). Since the *p*-value (0.0052) is lower than the commonly used threshold of 0.05, this indicates that there is now sufficient evidence to reject the null hypothesis, suggesting that there is a significant difference in participation by parental education level.

**Figure 2:**
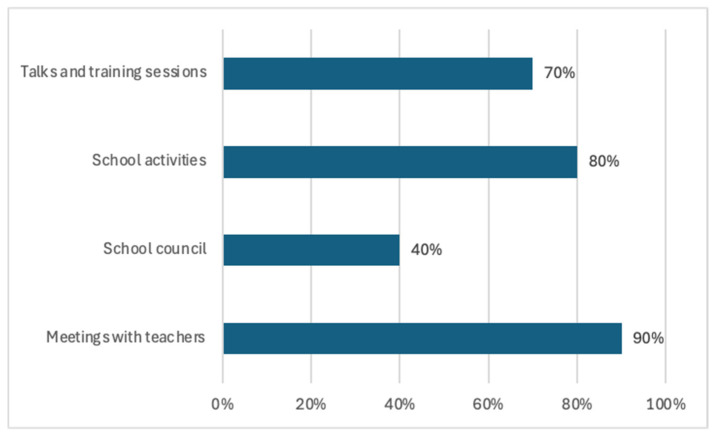
The involvement of parents in the different educational activities.

Despite the fact that parental participation in the different types of activities developed in schools not only benefits the academic and personal development of students, but also strengthens the school community and fosters an environment of collaboration and mutual support, the vast majority of respondents indicate that they tend to attend more meetings or tutorials with the tutor or specialist teachers (90%) than educational/cultural activities organised by the school, such as excursions, events, or school parties (80%). The former are considered more important because, as parents argue, it is the place where they are informed about their children’s school progress and the decisions taken at school. Other parents acknowledged belonging to the school council (40%) or attending information sessions that the school usually organises for their children’s education (70%), such as learning strategies, educational technology, and emotional development (Figure 2). Although the participation of families in the school council is low, it is considered not only a basic element for the success of pupils at school but also an indicator of the quality of the education system.

“Although I recognise the importance of all the activities organised by the school, I tend to be more involved in tutorials, as decisions are made that directly affect my child’s education and well-being”.

(INTERVIEW 15)

“Family participation in the school council remains low, due to the fact that some families are unaware of its functioning and importance”.

(INTERVIEW 18)

### 3.2. Support for Homework at Home

In relation to the frequency with which parents are involved in their children’s homework at home, it is worth noting that, as in the previous category, a large percentage of those interviewed (85.71%), state that they are very committed when their children have school activities at home, where parents help them to improve the teaching and learning process and solve those doubts and problems that may arise for their children with ASD. On the other hand, interviewees who denied this claim claimed that it is due to lack of time because of work and family reconciliation.

“I am quite active in that. I think it is an essential part of my role as a parent to help my son with his homework, especially because he has ASD. Whenever there are school activities at home, I make sure I am available to support him”.

(INTERVIEW 07)

“I really wish I could do more. I work full time and often have to bring work home, which limits the time I can spend helping with homework. I understand the importance of this support, especially because my daughter has ASD and needs more personalised attention”.

(INTERVIEW 31)

As in the previous category, it was found to be necessary to analyse whether parents’ educational level is associated with homework support.

The data suggest a significant difference in homework help according to parents’ educational level (Table 4). Parents with higher education tend to participate more (help) than those without higher education. To confirm whether these differences are statistically significant, the chi-square test was performed. The test score is 6.35 (sig. 0.0117). Since the *p*-value (0.0117) is lower than the commonly used threshold of 0.05, this indicates that there is sufficient evidence to reject the null hypothesis, suggesting that there is a significant difference in participation in household chores according to parents’ educational level.

Addressing the types of strategies that parents use to increase the motivation of their children with ASD in their studies, it should be noted that they use different methodologies to achieve an improvement in the teaching and learning process of their children. These include structuring the learning environment, where parents often create dedicated and structured spaces in the home that are predictable and free from distractions; the use of positive reinforcement, as this strategy involves the consistent use of rewards and praise to reinforce desirable study-related behaviours; and to a lesser extent, the use of information and communication technologies to enhance understanding and interest.

“We use a lot of visual aids because I have noticed that they capture their attention and really help them understand the concepts better. We also set up a regular schedule which seems to help him feel more confident and we eliminate distractions such as mobiles”.

(INTERVIEW 21)

“We introduced technology tools that he used at home into the classroom as well, and we saw a noticeable improvement in his concentration and participation. I think this alignment between home and school really helped create a more effective and comfortable learning environment for him”.

(INTERVIEW 01)

### 3.3. Communication and Collaboration

Regarding the use of different channels of communication between families and the school, it should be noted that the interviewees stated that they prefer to communicate via e-mail, as it is a more convenient and quicker means of communication. However, they mentioned that depending on the importance and seriousness of the situation, it is sometimes better to have a face-to-face meeting or tutoring.

“For me, email is direct and efficient. I can write down my questions or concerns and send them when I have time during the day. It also allows me to have a written record of all communications, which is useful for tracking progress and issues discussed”.

(INTERVIEW 31)

Regarding support groups for parents of children with ASD, 95.24% of the participants recognise that they participate in these groups as they offer resources, information, and a space to share experiences, mainly highlighting autism associations or early intervention centres.

“We have been members of the Seville Autism Association for ten years and we have found a lot of support in the meetings we have attended”.

(INTERVIEW 22)

“We take our daughter to the Early Attention Centre where she receives therapy and they have given us many tools to work with our daughter at home”.

(INTERVIEW 02)

### 3.4. Challenges and Suggestions

In terms of the main challenges encountered by parents who have children with ASD during their educational process, three main reasons were identified during the analysis of the interviews. These are related to the scarcity of resources on the part of the school in terms of adapted teaching materials, the ineffective communication that can sometimes occur between the school and the family, and the lack of awareness and training that can occur about the particularities of a pupil with ASD.

“Communication between school and families is crucial and often insufficient. We need to ensure that there is an open and effective line of communication so that parents feel part of the educational process”.

(INTERVIEW 09)

“A big problem is the lack of resources in the school; there are simply not enough trained staff and materials adapted for it. This has led me to look for external solutions such as finding specialised educational materials that we can use at home”.

(INTERVIEW 39)

On the other hand, addressing strategies and suggestions that can be made to address these challenges, it is worth noting that interviewees considered it necessary to carry out training programmes for the whole school community on the educational implication of ASD. On the other hand, it was also pointed out that the school should invest more in this issue and create a support network with other aware parents and teachers in order to provide efficient experiences and strategies.

“I consider it essential to invest more money in educational materials adapted for students with ASD. Nowadays there are insufficient resources in a school”.

(INTERVIEW 02)

“There is a need for greater awareness of the particularities of a pupil with ASD in the classroom. Therefore, the creation of training and awareness plans would be a way to help these students”.

(INTERVIEW 18)

## 4. Discussion

The analysis of the interviews has allowed us to explore the research questions formulated in this study. In relation to the first research question, which focuses on how parental involvement and support influences the academic performance of students with ASD, we find that parents with university studies tend to participate more in school activities and help with homework than parents without university studies; this may be mainly due to a lack of time because of their jobs or because of the family’s awareness of the importance of their involvement in the educational process of their children. This is evidenced by a number of previous studies that have shown the importance of parents’ involvement in their children’s education and that it is fundamental for the improvement of certain essential competences. Numerous studies have shown a positive relationship between parental involvement and the academic performance of students with ASD. Active parental involvement is associated with improvements in academic performance, social skills, and emotional adjustment. In this sense, parental involvement in the education of their children with ASD correlates with better academic performance and a greater likelihood of long-term educational success. In addition to academic achievement, parental involvement contributes to the holistic development of the student, including social, emotional, and psychological development. Students whose parents are actively involved tend to show higher levels of self-efficacy and general well-being [18,19]. Parental involvement and support in the educational process of students with ASD is essential for their academic success and holistic development. Collaboration between parents, teachers, and other professionals, as well as social support, are crucial to maximise the benefits of this involvement [5,20].

Addressing the second research question, more specifically which family strategies and practises contribute most to the school motivation of students with ASD, it should be noted that creating a structured learning environment at home can increase the motivation and academic achievement of students with ASD. Study participants emphasise the creation of dedicated study spaces, the implementation of regular timetables, and the elimination of distractions to improve their children’s education. According to Knight et al. [21], predictable and distraction-free environments help students with ASD to concentrate better and improve their academic performance. Furthermore, using positive reinforcement, such as praise and rewards to motivate students with ASD is a widely recognised practise. Consistent use of positive reinforcement can encourage desirable behaviours and improve the participation and interest of students with ASD in academic activities [22]. Finally, the use of ICT can help to engage the interest and enhance the understanding of students with ASD. These technologies, which include educational applications, specialised software, and adaptive devices, facilitate personalised learning tailored to their needs [23].

In relation to the challenges that families of children with ASD face in their involvement and participation in the educational process, thus answering the third research question, these include a shortage of adapted teaching resources, ineffective communication with schools, lack of awareness and training on ASD, time constraints and family stress, and limited access to support services. Many schools lack adequate educational materials to address the specific needs of students with ASD, limiting learning opportunities [24]. Poor communication between parents and teachers can lead to misunderstandings and a lack of cohesion in educational strategies [25]. Many families lack adequate training to understand and address the needs of their children with ASD [26]. This is coupled with time constraints, due to work responsibilities, which is a challenge for families of students with ASD, who often require increased attention and support [27]. Addressing these challenges requires joint efforts between schools, communities, and public policy to provide a more inclusive and effective supportive environment for these students and their families, as parental involvement in homework and educational activities at home is crucial to motivate and improve the academic performance of students with ASD [28]. At the same time, it is essential to mention that parents commonly experience high levels of stress, anxiety, and fatigue. This continuous state of stress and worry can significantly diminish parents’ quality of life, restricting their ability to perform adequately both personally and professionally [9]. For this, it is essential that parents have a support group and can access programmes specific to their children’s educational needs.

Finally, addressing the fourth research question, we can establish that parents of students with ASD perceive communication and collaboration with the school as a critical aspect of their children’s education, although there are diverse experiences and perceptions in this regard. Some parents report positive experiences, highlighting the importance of open, frequent, and effective communication for their children’s well-being and academic progress [25]. However, negative perceptions are also found among some parents, who feel that communication is ineffective or insufficient. These parents mention that the lack of clear and timely information can lead to frustration and a sense of isolation, negatively affecting their ability to adequately support their children [28].

In addition, parents highlight the need for more training and awareness-raising among school staff about the particularities of ASD. A lack of knowledge and understanding about ASD among educators can lead to misunderstandings and the implementation of ineffective strategies [29,30].

Parents have highlighted the need for greater training and awareness of school staff about the particularities of ASD. This lack of knowledge and understanding can lead to misunderstandings and the implementation of ineffective educational and social communication strategies for children with ASD [26]. In addition, insufficient staff training directly affects parents’ involvement in children’s social communication and limits the effectiveness of the services offered. Parents frequently find that available services do not adequately meet the specific needs of their children, highlighting a gap in the availability and appropriateness of services, including those offered online, which could be significantly improved with better informed and trained staff.

Before concluding, it is necessary to highlight important educational and social implications that need to be taken into account. Educationally, the need to increase parental participation in various school activities through flexible schedules is highlighted, as well as the importance of developing adapted teaching materials and providing specialised training for both educators and families. It is also recommended to employ personalised teaching strategies, including technologies and visual methods, to enhance the educational experience of students with ASD. Socially, we emphasise the need to reduce stigma and raise awareness of ASD, strengthen the school community through active parental involvement, and provide more support and resources for families. In addition, it is suggested to promote more flexible work policies to help parents balance their work and family responsibilities.

## 5. Conclusions

Parental involvement not only improves academic performance but also contributes significantly to students’ emotional well-being and motivation. The critical role of parental involvement in the education of children with ASD is highlighted, pointing out how parental involvement not only improves academic performance but also contributes significantly to students’ emotional and social well-being. The effectiveness of communication between parents and educational institutions is crucial, and while some parents report positive experiences, others perceive communication as insufficient. This underlines the need for improved communication and collaboration strategies to involve parents more effectively in the educational process. The results suggest that it is crucial to increase investment in resources and specific training on ASD for teachers and families, and the creation of support networks between parents and professionals as an effective strategy to share experiences and strategies that benefit students with ASD is also recommended. In addition, the importance of education policies that promote greater inclusion and the adequate allocation of resources to address diversity in classrooms is noted, highlighting the need for a collaborative and purposeful approach to ensure that all students have the opportunity to reach their full potential in an appropriately supported and accessible learning environment.

## 6. Limitations

Within the restrictions of this study, it is important to point out some limitations. First, due to the exploratory nature of the research and its focus on the perceptions of parents of students with ASD in secondary education in Seville, the results are not fully extensible to the whole country. In order to obtain a broader and more representative perspective, it would be valuable to carry out additional studies that include stratified samples from different autonomous communities and provinces in Spain. Furthermore, this research focuses exclusively on the perceptions of parents who have children with ASD. To achieve a more complete and detailed understanding, it would be beneficial to also include the perspectives of students and other education professionals. Incorporating these additional voices could provide new dimensions and significantly enrich the analysis. Finally, although we have endeavoured to conduct interviews with 42 parents, it would have been more beneficial to include a more representative sample, allowing us to gain insight into other important aspects of the research.

## 7. Future Directions

Future research directions on the impact of parental involvement on the educational development of students with ASD can focus on several key areas. Firstly, the identification of specific parental involvement strategies that are more effective, with the aim of improving the teaching and learning process of students with ASD through the application of child-friendly methodologies. Furthermore, research on variations in the effectiveness of parental involvement according to socio-economic, cultural, and contextual factors would be a very interesting topic and significantly related to the present study.

## Figures and Tables

**Table 1:** Distribution of participants according to socio-demographic aspects.

Category	Subcategory	Values	
**Gender**	Male	N	20
		%	47.6%
	Female	N	22
		%	52.3%
**Ownership of the centre** **(where your children are)**	Public	N	17
		%	40.4%
	Private-Concerted	N	14
		%	33.3%
	Private	N	11
		%	26.1%
**Years**	Between 30 and 40	N	13
		%	30.9%
	Between 40 and 50	N	23
		%	54.76%
	More than 60	N	6
		%	14.34%
**Educational level**	No higher education	N	13
		%	30.95%
	Higher education	N	29
		%	69.05%

Note: Grant-aided schools are those which are private educational institutions that have an agreement with the government to receive public funding. Public institutions are solely managed and funded by public bodies.

**Table 2:** Categorical system.

Categories	Subcategories	Description
**Participation in school meetings and activities**	Frequency	Frequency with which parents attend school meetings and activities.
	Type of activities	Types of school meetings and activities attended by parents.
**Homework support**	Frequency	How often do you help with your child’s chores at home?
	Support strategies and resources used at home	Specific strategies used to maintain or increase children’s motivation to study.
**Communication and collaboration**	Channels of communication	Means by which communication takes place (e-mail, meetings, reports, etc.).
	Participation in support groups	Participation in support groups or associations for parents of children with ASD.
**Challenges and suggestions**	Main challenges	Specific challenges parents have faced in the education of their children.
	Strategies or suggestions for overcoming challenges	Strategies and solutions that parents have implemented or suggest implementing to address these challenges.

**Table 3:** Participation according to parents’ educational level.

Overall Results of the Study		Breakdown of Participation	
Attendance	71.43% (n = 30)	No tertiary education	11.9% (n = 5)
		With higher education	59.52% (n = 25)
Non-attendance	28.57% (n = 12)	No tertiary education	19.05% (n = 8)
		With higher education	9.52% (n = 4)

**Table 4:** Involvement in homework according to parents’ level of education.

Overall Results of the Study		Breakdown of Participation	
Attendance	85.71% (n = 36)	No tertiary education	19.05% (n = 8)
		With higher education	66.67% (n = 28)
Non-attendance	14.29% (n = 6)	No tertiary education	11.9% (n = 5)
		With higher education	2.38% (n = 1)

## Data Availability

The data presented in this study are available upon request to the corresponding author. The data are not publicly available due to privacy restrictions.

## Appendix A

**Table A1:** Questions.

Q	Questions
1	Demographic questions: gender, age, ownership of your child’s centre.
2	How often do you attend school meetings or activities at school?
3	How do you support your child with homework at home?
4	What strategies do you use to maintain or increase your child’s motivation to study?
5	How is the communication between you and the school regarding your child’s academic progress and special needs?
6	Are you part of any support groups for parents of children with ASD?
7	What are the main challenges you have faced in your child’s education?
8	How have your own experience and expectations influenced your involvement in your child’s education?
9	What improvements would you suggest to better support students with ASD and their families?
10	Finally, is there anything else you would like to share?
